# Pattern of Facebook use by university students during the COVID-19 pandemic: relations with loneliness and resilience

**DOI:** 10.1007/s13278-023-01073-0

**Published:** 2023-04-05

**Authors:** Thanos Touloupis, Maria Sofologi, Dimitrios Tachmatzidis

**Affiliations:** 1 Department of Psychology, University of Western Macedonia, Florina, Greece; 2 Thessaloníki, Greece

**Keywords:** University students, Facebook intensity use, Self-disclosure, Resilience, Loneliness

## Abstract

Considering young adults’ extensive use of social media since the outbreak of the COVID-19 pandemic, the present study examined the pattern of Facebook use by university students during the period of hygienic crisis. Specifically, it was investigated students’ Facebook intensity use and self-disclosure to unknown online friends, as well as the role of sense of resilience and loneliness in the manifestation of the above Facebook behaviors. Overall, 792 undergraduate and postgraduate university students (48% women) completed online self-report questionnaires regarding the above variables. Undergraduate students, regardless of gender and Department of studies, made more intense Facebook use and self-disclosure to unknown online friends. Sense of loneliness positively predicted students’ online self-disclosure not only directly but also indirectly through their Facebook intensity use. Students’ resilience negatively moderated the relationship between sense of loneliness and Facebook behaviors. The findings propose a new explanatory model of emotional and behavioral mechanisms, which leads to a less safe pattern of Facebook use. This pattern possibly reflects youth’s collective tendency to use this social media platform recklessly as a way out of crisis periods, such as the pandemic period. The emergence of this pattern could be useful for launching or enriching university counselling/prevention actions aimed at strengthening students’ psycho-emotional skills, and subsequently their prudent use of social media.

## Introduction

Although today various social media are available to young people for free use, Facebook constitutes the most popular and attractive social networking site with almost 2 billion daily active users worldwide (Facebook 2022). Compared to other social networking sites, such as Instagram, Twitter, and TikTok, the platform of Facebook offers users more opportunities for multidimensional interactive communication (e.g., posting detailed information about themselves on their “wall”, posting audio-visual material on their online friends’ profile, group chatting, and fan pages). These characteristics make Facebook, compared to other social media, a fertile environment for intense social interaction in different ways (Kowal et al. 2020; Yeo and Ting 2017) and my explain why it is considered the most frequent tool of online communication among youth before and amid the period of the COVID-19 pandemic (Cunha et al. 2016; Duimel and DeHaan 2007; Hashim et al. 2020). Generally, it is worth mentioning that the majority (87%) of Facebook users are young adults (18–29 years old) during their academic life (Dritsas 2020; Duggan 2015; Statista 2019), who perceive it as an integral part of their daily lives, due to its significant academic and socio-interpersonal benefits (Lee and Winzenried 2009; Nikolopoulou and Gialamas 2016; Sciara et al. 2021; Urhahne et al. 2009).

Although Facebook use has been extensively investigated among adolescents, who use it to satisfy their developmental needs (tendency to experimentation, identity search, dating) (Crosslin and Golman 2014; Wang et al. 2018; Zalaquett and Chatters 2014), another important transitional period in a person’s life is the transition from school to academic life. During this period students must adapt to new and potentially challenging circumstances, such as setting up in a new city, independent living, the burden of academic obligations, and the establishment of new interpersonal relationships (Arnett 2007, 2014; Baggio et al. 2017). Therefore, it could be argued that university students’ familiarization with new situations can make academic years a challenging period during which students are likely to use social media and especially Facebook as a means of escaping from daily concerns and/or engaging new relationships. Τhe above challenges combined with the unexpected difficult situations related to the COVID-19 pandemic that have emerged in our lives, have led to a new research question: What is the collective pattern of university students’ behavior on social platforms and especially on Facebook during the unstable pandemic period?

The present study attempts to answer this research question focusing on university students in Greece where, as in other countries worldwide, tertiary education and academic life have been significantly affected by the measures against the COVID-19 pandemic (Bassett 2020; Mitsakis and Mahtab 2022).

### Facebook use by university students: literature gaps

Our attempt to investigate the pattern of university students’ behavior on Facebook platform during the COVID-19 pandemic arose because of specific literature gaps. Although Facebook use by university students has been thoroughly investigated by researchers in the past regarding its socio-interpersonal and academic benefits (Chuang and Liao 2021; Ellison et al. 2007; Zhang 2017), as well as the time spent on Facebook and the frequency of its use (e.g., Junco 2012; Locatelli et al. 2012; Michikyan et al. 2015; Sun 2020; Tandoc Jr et al. 2015), other more complex aspects of youth’s behavior on Facebook have been scarcely examined internationally. For example, Facebook is not a unidimensional site, but allows people’s active emotional over-engagement in multiple activities/applications reflecting in this way the intensity of its use. Another aspect of Facebook behavior is self-disclosure, which means that a person can share with great ease personal information, ideas, emotions, and activities with other Facebook friends met on Facebook (unknown online friends) via messaging and/or posting photos, videos, and self-descriptions (Boyd and Ellison 2007). According to some studies conducted on adolescents, it seems that there is a positive predictive relationship between the intensity of Facebook use and self-disclosure to unknown online friends. In other words, the more intense the use of Facebook, the more likely the student is to disclose personal sensitive information to Facebook friends met on Facebook (Dimogiorga and Syngollitou 2015; Tidwell and Walther 2002; Valkenburg and Peter 2009).

These specific aspects of Facebook use (intense use, self-disclosure) could reflect a less safe pattern of behavior on Facebook. This lies in the fact that Facebook use and, more specifically, the self-disclosure to unknown online friends on this platform has been found by some researchers to be related with problematic internet use (Kittinger et al. 2012) and attract the attention of online perpetrators, increasing in that way the likelihood of being victimized online (Barlett et al. 2018; Dredge et al. 2014; Rachoene and Oyedemi 2015).

Furthermore, the outbreak of the COVID-19 pandemic and the subsequent reduced people’s face-to-face social interaction (Bourdas and Zacharakis 2020; Galanis et al. 2020) seemed to have a negative impact on university students’ pattern of social media use, as even today they tend to use them excessively to arrange academic issues (e.g., projects) that were previously settled offline and to communicate with each other (Aristovnik et al. 2020; Burgess and Sievertsen 2020; Erliza and Septianingsih 2022). Within this context, it would be interesting to investigate whether, during the transitional period of academic life and especially amid the vulnerable period of the COVID-19 pandemic, university students tend to adopt a collectively maladaptive pattern of Facebook use by making intense use of it and self-disclosure to unknown online friends. This finding could highlight that during unstable periods preventing their intense Facebook use may protect them from other online behaviors (self-disclosure to unknown people) related to unpleasant experiences (e.g., cyberbullying) This research question seems to remain unanswered to date, as most of the current studies of the last two years do not focus on university students but on adults in general, examining just their time spent on social media (Eghtesadi and Florea 2020; Indriawati and Wibowo 2021; Raamkumar et al. 2020; Wiederhold 2020) and not the specific aspects of Facebook use under the present study.

### The role of loneliness and resilience in Facebook use

Examining university students’ collective pattern of Facebook use amid the ongoing pandemic period, we could not omit to examine, not only the specific behaviors described in the previous subsection, but also the contribution of psycho-emotional factors in the development of this pattern of Facebook use. According to the literature, these factors among others include sense of loneliness and resilience (Alheneidi et al. 2021; Bilgin and Tas 2018; Costa et al. 2019; Κim et al. 2009; Yen et al. 2019). Sense of loneliness constitutes an emotional variable that has reached research attention during the period of the COVID-19 pandemic. Sense of loneliness relates to someone’s perceived sense of social isolation due to the difference between the desired and actual social relationships (Holt-Lunstad et al. 2015). On the other hand, resilience is defined as an individual’s ability to adapt positively to a situation, despite difficult and adverse conditions and despite exposure to risk factors (Fergus and Zimmerman 2005; Luthar 2006; Masten 2001; Masten and Narayan 2012; Rutter 2006; Zhou et al. 2017). It is considered that in early adulthood, which is usually identified with the period of academic life, individuals usually have built up some capacity, such as resilience, to deal with adversities and they do not need to rely anymore on their parents for their regulation of well-being. In other words, young adults, such as university students, are supposed to have built up a generalized sense of resilience, which could be perceived as a protective filter against possible predicaments (Arnett 2007, 2014; Connor and Davidson 2003).

Based on related studies, people’s sense of loneliness seems to positively predict their over-use of internet and social networking sites (Alheneidi et al. 2021; Costa et al. 2019; Κim et al. 2009). In other words, the higher individuals’ sense of loneliness, the more likely they are to use internet and social media in a problematic (intense) and possibly unsafe way (self-disclosure to unknown online friends) (Alheneidi et al. 2021; Costa et al. 2019; Κim et al. 2009). These findings along with the positive predictive relationship between intense Facebook use and self-disclosure to unknown online friends (Dimogiorga and Syngollitou 2015; Tidwell and Walther 2002; Valkenburg and Peter 2009), as it was described in the previous subsection, could make us consider the intensity of Facebook use as a mediator between sense of loneliness and self-disclosure to unknown online friends. Namely, when people make intense use of Facebook, this can burden the impact of their sense of loneliness on making self-disclosures to unknown online friends. In other words, loneliness may result in intense use of Facebook, which in turn increases the likelihood of making self-disclosures to unknown online friends.

Although people’s sense of loneliness can have an impact on the development of a less safe collective pattern of Facebook use, such as making intense use of it, it does not influence all people equally. Based on the protective factor framework (Masten 2001), the presence of a protective factor such as resilience may significantly decrease the likelihood of developing a dysfunctional behavior on Facebook (Bilgin and Tas 2018; Nam et al. 2018; Yen et al. 2019), and therefore ameliorate/buffer the negative impact of loneliness on the intensity of Facebook use. Consequently, resilience was considered as a variable which can moderate the impact of loneliness on the intensity of Facebook use.

**Fig. 1:**
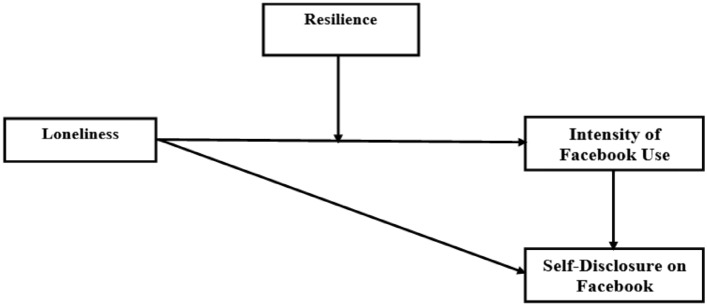
Hypothetical structural model of the network of relationships among variables (*N* = 792)

Based on the above, a structure of relationships among the variables could be expected, according to which loneliness may positively predict self-disclosure to unknown online friends not only direct but also indirect through the mediating role of intensity of Facebook use. Additionally, resilience may moderate the relationship between loneliness and intensity of Facebook use. The theoretical structural model of the variables of the present study is illustrated in Fig. 1.

According to the authors’ knowledge, no study to date has identified in the context of a holistic approach, this structural model of relationships between emotional (loneliness, resilience) and behavioral variables (intensity of Facebook use) that better explain self-disclosure to unknown online friends especially during academic life. The necessity to investigate the above explanatory model in a university student population is even more pronounced in the period of the COVID-19 pandemic, as amid and in the aftermath of this hygienic crisis young adults, regardless of their gender, tend to report a high sense of loneliness and a low sense of resilience (Ernst et al. 2022; Groarke et al. 2020; Hwang et al. 2020; Rosenberg 2020; Vinkers et al. 2020; Zhang et al. 2022).

### Facebook use: do department, level of studies, and gender matter?

Except for the role of psycho-emotional factors, there is very little and/or conflicted knowledge about whether students’ pattern of Facebook use (intense use, self-disclosure to unknown online friends) is differentiated based on personal/demographic characteristics, such as their university department, level of studies, and their gender.

For example, most of the related studies have been conducted on a mixed sample of students who attend Polytechnic Schools, as well as Schools of Humanities and Health Sciences (Anand et al. 2018; Younes et al. 2016). Consequently, there are no comparative findings regarding the Facebook experiences under study (e.g., intensity of use and self-disclosure to unknown online friends) among students from different university Departments. This would be interesting considering that students from Schools of Humanities and Social Sciences (e.g., Departments of Psychology/Education), according to their Guide of undergraduate/postgraduate studies, are expected to be taught and be familiar with issues that relate to human risky behaviors (e.g., excessive use of social networking sites), as well as protective/risk factors (Guide of Undergraduate/Postgraduate Studies, Department of Psychology/Education, University of Western Macedonia, 2021–2022). Therefore, these students would be expected to be more sensitized toward a safer pattern of Facebook use, compared to students from other university Schools. Adopting the above comparative approach in a future study, we could highlight possibly different needs between the university Departments in the implementation of counselling/prevention actions aimed at strengthening students’ safe use of social media.

Furthermore, regarding the level of studies, the available studies (Anand et al. 2018; Younes et al. 2016) do not investigate possible differences between undergraduate and postgraduate students regarding their pattern of Facebook use. Considering that undergraduate and postgraduate programs of studies imply different obligations and orientations at academic level (Guides of Undergraduate/Postgraduate Studies, Department of Psychology/Education, University of Western Macedonia, 2021–2022), as well as a possibly different proximity of interpersonal relationships, a research question arises: Could these qualitatively different contexts and circumstances of undergraduate and postgraduate studies make students adopt a different perspective and pattern of behavior on Facebook? The present study, trying to answer this question, could possibly highlight university student subgroups that should be more involved in university awareness-raising activities on safe online culture.

As far as gender’s effect on Facebook use, the related studies have been primarily conducted on adults rather than university students leading to mixed findings. For example, some studies mention men’s extensive Facebook use (Durndell and Haag 2002; Joiner et al. 2005; Raacke and Bonds-Raacke 2008; Vigna-Taglianti et al. 2017), while others associate this behavior mainly with women (Hargittai 2007; Sheldon 2008). Also, compared to men, women more often make self-disclosure on social networking sites (Farinosi and Taipale 2018; Peter et al. 2005; Petronio 2002; Rose and Rudolph 2006; Sheldon 2013), as they mention that during online communication they share more familiar and personal information with online strangers (Jackson et al. 2001; Li 2006; Special and Li-Barber 2012; Stern 1999, 2002a, 2002b). The above findings are consistent with attitudes stereotypically related to female gender in offline interpersonal communication, as previous studies have shown that women seem to be more oriented toward the development and maintenance of interpersonal relationships (Eagly 1987). On the other hand, however, other studies conclude that men are more likely to make self-disclosure to make new acquaintances and develop new relationships with unknown online friends (Cudo et al. 2020; Mazman and Usluel 2011; Muscanell and Guadagno 2012). Therefore, based on the above mixed findings, we cannot safely conclude whether women or men university students are more prone to develop more intense Facebook use, in the context of which they may succumb to the temptation to make self-disclosures to unknown online friends regarding personal and sensitive information. Trying to clarify this we could inform about gender-based risk groups of university students who tend to adopt a less safe pattern of Facebook use.

### Purpose, goals and hypotheses of the present study

Based on the above literature review, the present study aimed to investigate the pattern of Facebook use by university students considering their perceived intensity of Facebook use and self-disclosure to unknown online friends during the period of the COVID-19 pandemic. At the same time, the role of students’ sense of loneliness and resilience in the development of the above pattern of Facebook use was examined. Specifically, the following research questions emerged:

Do university students make intense Facebook use and self-disclosure to unknown online friends during the period of the COVID-19 pandemic?
What is the structure of relationships between emotional (loneliness, resilience) and behavioral variables (intensity of Facebook use) that better explain self-disclosure to unknown online friends?
(a) Do students’ Department, of studies (b) level of studies (undergraduate/postgraduate) and (c) gender differentiate their intensity of Facebook use and their self-disclosure to unknown online friends?

According to the related literature, the following were expected:

1. During the period of the COVID-19 pandemic university students make intense Facebook use and self-disclosures to unknown online friends (Hypothesis 1) (Junco 2012; Locatelli et al. 2012; Michikyan et al. 2015; Tandoc Jr et al. 2015).
2. University students’ sense of loneliness positively predicts, their self-disclosure to unknown online friends through the positive mediating role of the intensity of Facebook use (Hypothesis 2a) (Alheneidi et al. 2021; Costa et al. 2019; Dimogiorgia and Syngollitou 2015; Κim et al. 2009; Tidwell and Walther 2002; Valkenburg and Peter 2009). Students’ sense of resilience negatively moderates the relationship between their sense of loneliness and their intensity of Facebook use (Hypothesis 2b) (Alheneidi et al. 2021; Bilgin and Tas 2018; Costa et al. 2019; Dimogiorgia and Syngollitou 2015; Κim et al. 2009; Nam et al. 2018; Tidwell and Walther 2002; Valkenburg and Peter 2009; Yen et al. 2019).
3. Regarding the effect of the students’ Department and their level of studies (undergraduate/postgraduate) on their intensity of Facebook use and their self-disclosures to unknown online friends, we are not allowed to make related Hypotheses due to the lack of related research findings. Finally, based on the conflicted findings (e.g., Cudo et al. 2020; Jackson et al. 2001; Mazman and Usluel 2011; Muscanell and Guadagno 2012; Rose and Rudolph 2006; and Sheldon 2013), it is expected that university students’ gender does not differentiate their Facebook behaviors under study (intensity of Facebook use, self-disclosure) (Hypothesis 3).

## Materials and method

### Sample

The pilot sample of the study involved 102 university students [58 (57%) women, 44 (43%) men] from different Departments of Greek Universities, who completed a set of online self-report questionnaires. The pilot study did not indicate the need to modify the questionnaires. Therefore, the pilot sample (*N* = 102) was integrated into the sample of the main study (*N* = 690 students) resulting in a total sample of 792 university students [380 (48%) women, 412 (52%) men]. Out of them, 59% were undergraduate and 41% were postgraduate (MSc, PhD, Postdoc) students. Most of the undergraduate students were in the second year of their studies (70%), while most of the postgraduate students (85%) attended a Master (MSc) program. Due to this unequal distribution, we were not statistically allowed to examine the effect of the year of undergraduate studies, or the effect of the postgraduate program attended (MSc, PhD, Postdoc) on the variables under study. As a result, the sample is presented in two broad levels of studies (undergraduate and postgraduate). Regarding the Department of studies, students studied in Departments of Schools of Humanities and Social (37%), Polytechnic (30%), and Economic Sciences (33%). Finally, regarding the age of the students (*Μean* = 23, SD = 4.70), 120 (42%) were 20 years old, 102 (35%) were 24 years old, 30 (10%) were 21 years old, 15 (5%) were 28 years old, 12 (4%) were 22 years old, 10 (3%) were 19 years old, and 3 (1%) were 34 years old.

### Instruments

For the purpose of the present study, a set of online self-reported questionnaires was used. After the introductory demographic questions (e.g., gender, age, and level/Department of studies), four main parts followed:

*Facebook Intensity Use Scale*: Students’ perceived intensity of Facebook use was measured with the Greek translation of the Facebook Intensity Use Scale (Ellison et al. 2007), which has been previously used in a sample of Greek adolescent students with good psychometric properties (*α* = .83) (Dimogiorga and Syngollitou 2015). This scale was selected due to the fact that it is the only one in international literature that evaluates the use of social networking sites, such as Facebook, including questions and statements that measure not only the frequency of its use but also the extent of students’ active involvement in the activities of Facebook. The scale evaluates: (a) the number of Facebook friends based on an eight-point scale (from 1 = *up to 10 friends* to 8 = *over 400 friends*) and (b) the time spent on Facebook per day based on a five-point scale (from 1 = *up to 10 min* to 5 = *over 3 h*). Furthermore, the scale includes six statements to tap the extent to which participants are emotionally connected to Facebook and the extent to which Facebook is integrated into their daily activities (e.g., “Facebook is part of my daily routine activity”, “I am proud when I tell others that I have Facebook profile too”), reflecting in that way the intensity of Facebook use. Participants are asked to respond to the statements based on a five-point Likert scale, ranging from (1) = *not valid at all* to (5) = *absolutely valid*. According to the initial constructors of the scale (Ellison et al. 2007), intensity of Facebook use can be reflected not only in a qualitative way, such as an individual’s emotional connectedness to Facebook activities (e.g., chatting and posting), but through quantitative dimensions as well, such as the number of Facebook friends and the time spent on Facebook daily. In this way, the scale tries to reflect a multidimensional and more generalized sense of an individual’s intensity of Facebook use. The index of Facebook Intensity Use derives from the average of the total score of the questions and the statements, as long as they have been previously converted to standardized *z* scores due to the differing item scale ranges. Based on this standardization, the cutoff scores range from 8 to 48 points with 24 points (Mean = 2.4, SD = 1.21) corresponding to an average intensity of Facebook use. The higher the score (and the corresponding Mean) the higher the intensity of Facebook use (see Ellison et al. 2007).

In order to test the factorial validity of the scale in the university student sample, a principal component analysis was carried out using the main component method and Varimax-type rotation (KMO = .892, Bartlett Chi-square = 1982.133, *p* < .001). One factor emerged with eigenvalue > 1.0 and significant interpretive value (Table 1), confirming the unidimensional structure of the scale (Dimogiorga and Syngollitou 2015; Ellison et al. 2007): Factor 1 = Intensity of Facebook use, explaining 51.22% of the total variance. The internal consistency index for the factor is *α* = .823. The affinities (according to Pearson’s correlation coefficient r) of the score of each question of the factor with the sum of the scores of the remaining questions of the factor (corrected item—total correlation) are considered satisfactory [in a sample of 300 and 600 people, loadings of more than .29 and .21, accordingly, are accepted (Field 2005)]: Factor 1 (from *r* = .47 to *r* = .83).

**Table 1:** Principal component analysis of the Facebook intensity use scale (*N* = 792)

Questions/Statements	F1
1. About how many total Facebook friends do you have?	.488
2. On average, approximately how many minutes per day do you spent on Facebook?	.512
3. Facebook is part of my everyday activity	.733
4. I am proud to tell people I’m on Facebook	.551
5. Facebook has become part of my daily routine	.872
6. I feel out of touch when I haven’t logged onto Facebook for a while	.719
7. I feel I am part of the Facebook community	.698
8. I would be sorry if Facebook shut down	.544

F1: Factor “Intensity of Facebook Use”

All of the above standardized loadings of the factor are statistically significant (*p* < .05)

*Self-Disclosure Index*: Students’ self-disclosure to Facebook friends met on Facebook (unknown online friends) was examined with the Greek translation of the Self-disclosure Index (Miller et al. 1983), which has been previously used in a sample of Greek adolescent students with good psychometric properties (*α* = .92) (Dimogiorga and Syngollitou 2015). The “Self-Disclosure Index”, which includes ten statements, is the only one in the related literature measuring the frequency at which a person reveals personal information on various subjects (e.g., “I reveal my personal interests and hobbies”, “I reveal my deepest feelings”) on Facebook friends met on Facebook (unknown online friends). Responses are given on a four-point Likert scale (from 1 = *never* to 4 = *often*). Individual items are summed to produce an overall score ranging from 10 to 40 points with 20 points (Mean = 2.0, SD = 1.09) corresponding to an average level of self-disclosure to unknown Facebook friends. Therefore, higher scores (and corresponding Means) indicate higher levels of self-disclosure on Facebook (see Miller et al. 1983).

In order to test the factorial validity of the Index in the university student sample, a principal component analysis was carried out using the main component method and Varimax-type rotation (KMO = .845, Bartlett Chi-square = 1911.409, *p* < .001). One distinct factor emerged with eigenvalue > 1.0 and significant interpretive value (Table 2) in-line with the original unidimensional structure, confirming the unidimensional structure (Dimogiorga and Syngollitou 2015; Miller et al. 1983): Factor 1 = Self-Disclosure, explaining 51.98% of the total variance. The internal consistency indexes for Factor 1 is *α* = .85. The affinities (according to Pearson’s correlation coefficient r) of the score of each question by Factor 1 with the sum of the scores of the remaining questions of the factor (corrected item—total correlation) are considered satisfactory: Factor 1 (from *r* = .39 to *r* = 59).

**Table 2:** Principal component analysis of the self-disclosure index scale (*N* = 792)

Statements/proposals	F1
1. I reveal my personal interests and hobbies	.841
2. I reveal things I've done for which I feel guilty	.772
3. I reveal things I wouldn't do in front of people	.689
4. I reveal my deepest feelings	.754
5. I reveal what I like and what I don't like about myself	.791
6. I reveal what's important to me in life	.819
7. I reveal what makes me who I am	.858
8. I reveal my worst fear	.701
9. I reveal things I've done, and I feel proud of	.599
10. I reveal my close relationships with other people	.801

F1: Factor “Self-Disclosure”

All of the above standardized loadings of the factor are statistically significant (*p* < .05)

*Resilience Scale*: Students’ resilience was examined with the Greek translation of the short version of the Connor-Davidson Resilience Scale (The Connor-Davidson Resilience Scale—CD-RISC; Connor and Davidson 2003) of Campbell-Sills and Stein (2007). The original long version of the CD-RISC (25 items) investigates individuals’ positive adaptation to stressful and/or difficult situations. According to Campbell-Sills and Stein (2007), the factor structure of the original CD-RISC across demographically equivalent samples is unstable, while the short version of the scale has good psychometric properties (*α* = .89) in a sample of undergraduate university students. This finding demonstrates that resilience can be reliably assessed with a subset of the CD-RISC items. Thus, in the short version of the CD-RISC, resilience is measured through ten representative statements/proposals (they reflect individuals’ ability to tolerate experiences such as change, personal problems, illness, pressure, failure, and painful feelings) which form the single factor “resilience” (Campbell-Sills and Stein 2007). These statements/proposals are answered on a 5-point Likert scale (from 0 = *not at all true* to 4 = *almost always true*). Examples of the statements/proposals are the following: “I tend to bounce back after illness or hardship”, “I can stay focused under pressure”, and “I am able to adapt to change”. Individual items are summed to produce an overall score ranging from 0 to 40 points with 20 points (Mean = 2.0, SD = 1.91) corresponding to an average level of resilience. Therefore, higher scores (and corresponding Means) indicate higher levels of resilience (see Campbell-Sills and Stein 2007).

In order to test the factorial validity of the scale, a principal component analysis was carried out using the main component method and Varimax-type rotation (KMO = .833, Bartlett Chi-square = 1499.311, *p* < .001). One distinct factor emerged with eigenvalue > 1.0 and significant interpretive value (Table 3) in-line with the original factor structure: Factor 1 = Resilience, explaining 44.12% of the total variance. The internal consistency indexes for Factor 1 is *α* = .811. The affinities (according to Pearson’s correlation coefficient *r*) of the score of each question by Factor 1 with the sum of the scores of the remaining questions of the factor (corrected item—total correlation) are considered satisfactory: Factor 1 (from *r* = .41 to *r* = 88).

**Table 3:** Principal component analysis of the resilience scale (*N* = 792)

Statements/proposals	F1
1. I am able to adapt to change	.502
4. I can deal with whatever comes	.599
6. I try to see humorous side of problems	.702
7. Coping with stress can strengthen me	.689
8. I tend to bounce back after illness or hardship	.581
11. I can achieve goals despite obstacles	.699
14. I can stay focused under pressure	.505
16. I am not easily discouraged by failure	.791
17. I thinks of self as strong person	.499
19. I can handle unpleasant feelings	.551

F1: Factor “Resilience”

All of the above standardized loadings of the factor are statistically significant (*p* < .05)

*Loneliness Scale*: Students’ sense of loneliness was examined with the Greek translation of the short form of California Los Angeles Loneliness Scale—UCLA-LS (ULS-8; Hays and DiMatteo 1987), which has been previously used in samples of university students with satisfactory psychometric properties (*α* = .72) (Doğan et al. 2011; Wu and Yao 2008). According to previous studies, there are concerns with the factorial validity of the original 20-item UCLA-LS, indicating that a short form is also reliable and valid as the original 20-item scale while also displaying superior model fit and reduce the burden on respondents (Doğan et al. 2011; Wu and Yao 2008). This scale includes eight proposals/statements, for which individuals are asked to state how often they feel as each proposal/statement describes on a four-point Likert scale ranging from (1) = *never* to (4) = *always*. Specifically, the scale consists of two positively worded (“I am an outgoing person,” and “I can find companionship when I want it”), which are reverse scored. Individual items are summed to produce an overall score ranging from 20 to 80 points. Based on these cutoff scores and considering the four-point Likert scale 40 points correspond to an average sense of loneliness (Mean = 2.0, SD = 1.09). The higher the total score of the scale (and the corresponding Mean) the higher the sense of loneliness (Hays and DiMatteo 1987). Examples of the proposals/statements are the following: “People are around me but not with me”, “I lack companionship”, and “There is no one I can turn to”.

In order to test the factorial validity of the scale, a principal component analysis was carried out using the main component method and Varimax-type rotation (KMO = .872, Bartlett Chi-square = 1443.144, *p* < .001). One factor emerged with eigenvalue > 1.0 and significant interpretive value (Table 4), confirming the unidimensional structure of Hays and DiMatteo (1987): Factor 1 = Loneliness, explaining 32.11% of the total variance. The internal consistency indexes for this Factor is *α* = .821. The affinities (according to Pearson’s correlation coefficient *r*) of the score of each question by the factor with the sum of the scores of the remaining questions of the factor (corrected item—total correlation) are considered satisfactory: from *r* = .48 to *r* = .58.

**Table 4:** Principal component analysis of the loneliness scale (*N* = 792)

Statements/Proposals	F1
1. I lack companionship	.598
2. There is no one I can turn to	.711
3. I am an outgoing person^a^	.742
4. I feel left out	.528
5. I feel isolation from others	.633
6. I can find companionship when I want it^a^	.701
7. I am unhappy being so withdrawn	.577
8. People are around me but not with me	.723

^a^Scores of reversed items have been recorded

F1: Factor “Loneliness”

All of the above standardized loadings of the factor are statistically significant (*p* < .05)

### Procedure

After the approval for the survey by the Research Ethics Committee (REC) at the University of Western Macedonia, the authors randomly selected Departments from different Schools (e.g., Humanities and Social Sciences and Polytechnic) of four Universities whose student population is considered representative of the whole university student population in Greece and in other European countries (Nadoveza Jelić and Gardijan Kedžo 2018). Subsequently, an email was sent to the secretariats of the selected Departments asking the announcement and the link of the study to be post on the websites of each Department. Apart from the details about the study and the identity of the researchers, the email included the relevant link to the questionnaire that was designed using the online Google Drive platform, as well as the attached approval of the REC. In the online announcement about the study, it was clarified that a necessary condition for students’ participation was to have a profile on Facebook. The answers of the 792 students who responded were automatically entered in a logistic sheet of the platform. The above process was carried out for both phases, the pilot, and the main phase of the study. The duration for the completion of the questionnaires was estimated at around 15 min. The participation of the students was voluntary, while all the criteria of anonymity and confidentiality of the data were met.

### Methods of analyses

For the present study, initially, the psychometric properties of the scales were tested through principal component analysis and Cronbach’s alpha (see subSect. 2.2). To depict university students’ perceived intensity of Facebook use and self-disclosure to unknown online friends (Hypothesis 1), as well as their sense of loneliness and resilience descriptive statistic was applied. To investigate the dyadic relations between the above variables a series of Pearson correlation analyses was carried out (Pearson *r*). The confirmation of the research hypotheses Hypothesis 2a and 2b was checked by applying path analysis to the data (using the Mplus programme with the Maximum Likelihood method) to depict the structure of the relationships between the variables involved, which leads to students’ self-disclosure to unknown online friends. To examine the effect of students’ Department and level of studies (undergraduate/postgraduate) on the Facebook behaviors under study (intensity of use, self-disclosure) one-way ANOVAs were carried out. Students’ Department and their level of studies (undergraduate/postgraduate) were set as the independent variable in the first and second analysis, respectively, while the Facebook behaviors (intensity of use, self-disclosure) were separately considered as the dependent variables in each case. The same analysis (ANOVA) was applied to investigate the effect of students’ gender on their Facebook behaviors (intensity of use, self-disclosure) (Hypothesis 3). In both ANOVAs gender was set as the independent variable while the Facebook behaviors (intensity of use, self-disclosure,) were separately considered as the dependent variables in each case.

## Results

### Students’ perceived intensity of Facebook use, self-disclosure to unknown online friends, loneliness and resilience

According to the results, most of the university students mentioned that they have over 400 Facebook friends (81%) and spend more than 3 h per day on Facebook (69%). Also, considering the cutoff scores of each scale, it seems that students expressed a relatively high (more than average) intensity of Facebook use (Mean = 4.11, SD = .57) as well as a relatively high (more than average) self-disclosure to unknown Facebook friends (Mean = 3.32, SD = .82).

Accordingly, based on the students’ self-reports, and considering the cutoff scores of each scale, it seemed that students experience a relatively high (more than average) sense of loneliness (Mean = 3.31, SD = .44) and a relatively moderate level of sense of resilience (Mean = 2.21, SD = .59).

### Correlations among Facebook behaviors, loneliness, and resilience

In Table 5, it is observed a positive correlation between students’ perceived intensity of Facebook use and their self-disclosure on online friends met on Facebook (*r* = .54, *p* < .01). On the contrary, students’ sense of loneliness and resilience seemed to negatively correlate with each other (*r* = − .43, *p* < .01). Students’ sense of loneliness seemed to have a positive correlation with both intensity of Facebook use (*r* = .46, *p* < .01) and self-disclosure to unknown Facebook friends (*r* = .14, *p* < .05), while students’ sense of resilience was negatively correlated with their Facebook behaviors mentioned above (intensity of Facebook use: *r* = − .33, *p* < .01, self-disclosure: *r* = − .14, *p* < .05).

**Table 5:** Correlations among variables (*N* = 792)

	1	2	3	4
1 Intensity of Facebook use				
2 Self-disclosure on Facebook	.54**			
3 Resilience	− .33**	− .14*		
4 Loneliness	.46**	.14*	− .43**	

*p < .05, **p < .01

No statistically significant correlations (*p* > .05) were omitted

### Path analyses among Facebook behaviors, loneliness, and resilience

**Fig. 2:**
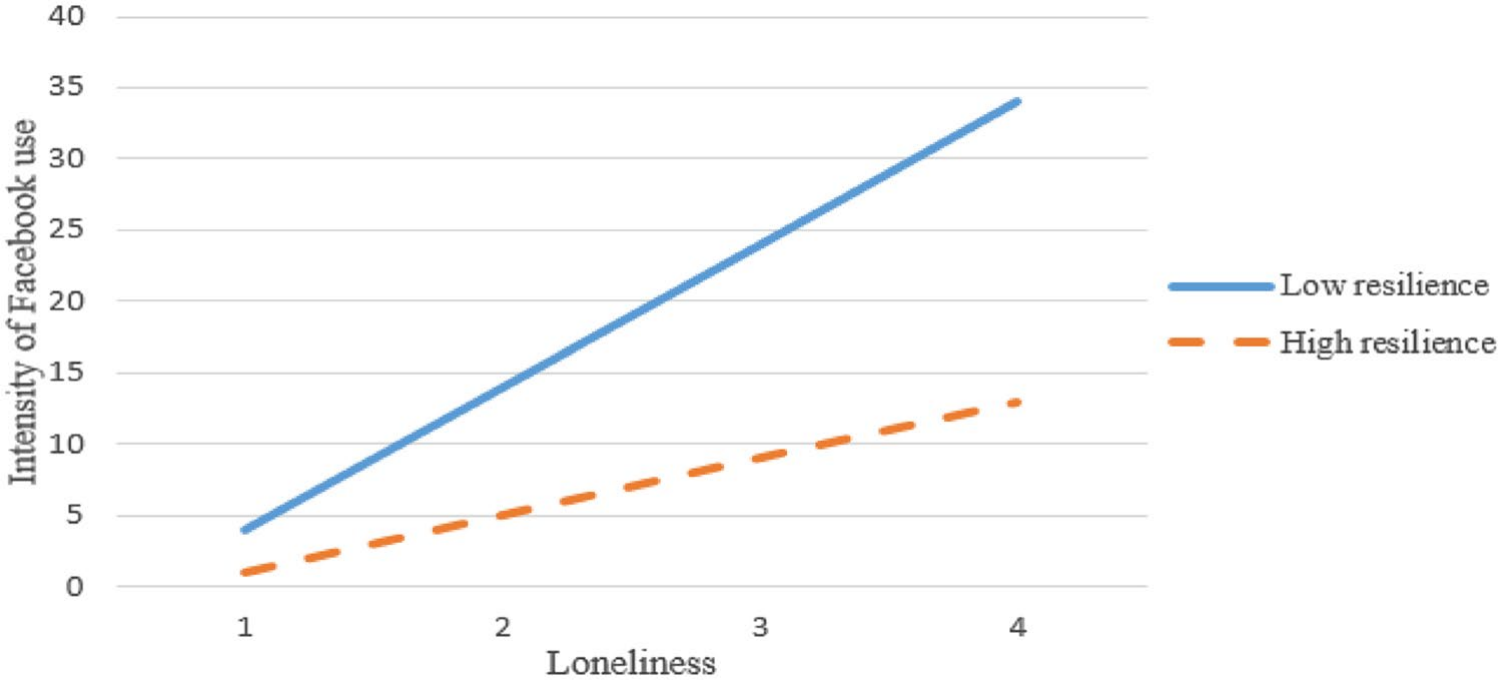
Influence of loneliness on intensity of Facebook use with higher and lower levels of resilience (*N* = 792)

To map the structure of the relationships (path analyses) between the variables involved (loneliness, resilience, intensity of Facebook use), which leads to the self-disclosure to unknown online friends (dependent variable), a series of preliminary analyses of multiple regressions was performed to check the dyadic predictive relationships between the variables. Specifically, according to these analyses, it was found that sense of loneliness positively predicts intensity of Facebook use (*R*^2^ = .139, *beta* = .32, *t* = 5.514, *p* < .01). Furthermore, intensity of Facebook use positively predicts self-disclosure to unknown online friends (*R*^2^ = .219, *beta* = .43, *t* = 5.811, *p* < .01). Also, sense of loneliness seemed to positively predict self-disclosure to unknown Facebook friends (*R*^2^ = .201, *beta* = .38, *t* = 5.722, *p* < .01). To test whether students’ sense of resilience moderates the relationship between their sense of loneliness and their intense Facebook use a hierarchical multiple regression analysis was conducted. In the first step, two variables were included: sense of loneliness and sense of resilience. These variables accounted for a significant amount of variance in students’ intensity of Facebook use, *R*^2^ = .340, *F*(2, 790) = 76.57, *p* < .001. Next, the interaction term between students’ sense of loneliness and their sense of resilience was added to the regression model, which accounted for a significant proportion of the variance in students’ intense Facebook use, Δ*R*^2^ = .02, Δ*F*(1, 788) = 9.27, *p* < .01, *beta* = − .432, *t*(288) =—2.813, *p* < .01. Examination of the interaction plot (Fig. 2) showed a buffering effect, such that the higher the sense of resilience the lesser the predictive effect of loneliness on intensity of Facebook use. On the other hand, lower sense of resilience pertained to more intense Facebook use with the increase of sense of loneliness.

**Fig. 3:**
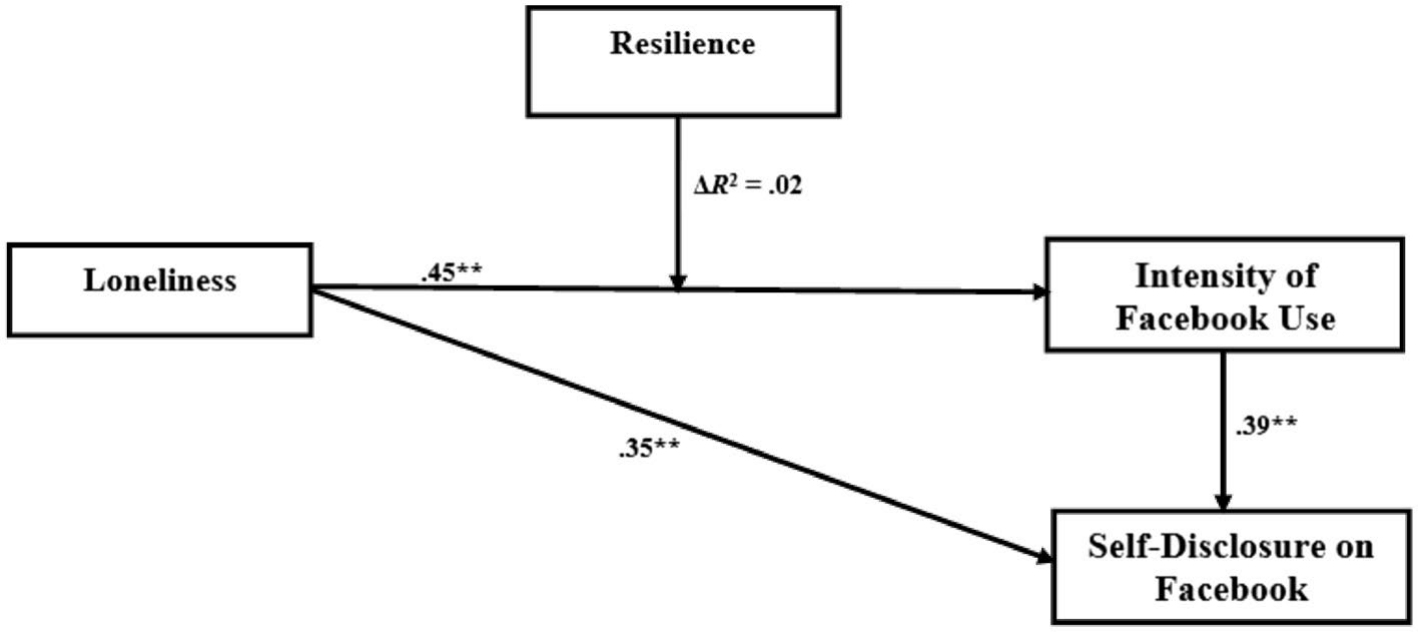
Schematic representation of the path model for the students’ self-disclosure on Facebook (*N* = 792). The values on the arrows are standardized coefficients of the model. **p < .01

Meeting the assumptions of normality, the above preliminary regression analyses showed that all the variables had significant predictive relationships between them. Therefore, all the variables of the study were included in the path analyses. Without any missing cases, the path model that emerged from the students’ responses had good fit indexes: *χ*^2^ (29, *Ν* = 791) = 45.134, *p* > .05 (CFI = .981, TLI = .929, RMSEA = 0.063, SRMR = 0.084) (Fig. 3).

In the structure of the relationships (Fig. 3), the path diagram illustrates the regression relationships between the variables. Sense of loneliness constitutes the exogenous variable, which predicts the other variables, without being predicted by other variables. From the rest of the variables, which are endogenous, one is considered dependent (self-disclosure to unknown Facebook friends) and one is viewed as a moderator (resilience). According to Fig. 3, students’ sense of loneliness predicted directly and positively their perceived intensity of Facebook use (*beta* = .35, *p* < .01). That is, the higher the students’ sense of loneliness the more likely they are to feel emotionally connected to Facebook making intense use of it. Accordingly, it was found that students’ perceived intensity of Facebook use predicts in a direct and positive way their self-disclosure to unknown online friends (*beta* = .43, *p* < .01). That is, the more intense the Facebook use by students the more likely they are to make a self-disclosure to unknown Facebook friends. Also, students’ sense of loneliness seemed to predict directly and positively their self-disclosure to unknown Facebook friends (*beta* = .29, *p* < .05). Therefore, based on standardized normal distribution value (*Z*), it seemed that sense of loneliness positively predicts self-disclosure to unknown online friends not only directly but indirectly as well through the mediating role of intensity of Facebook use (*Z* = 2.45, *p* < .05).

Furthermore, the path diagram (Fig. 3) showed that students’ sense of resilience moderates negatively the relationship between their sense of loneliness and their perceived intensity of Facebook use (*Z* = -2.34, *p* < .05). In other words, students’ sense of resilience may decrease the likelihood students who feel lonely to make intense Facebook use.

### The effect of students’ department, level of studies and gender on Facebook behaviors

Meeting the assumptions of Levene’s Test of Equality of Error Variances for both Facebook behaviors (intensity of use: *F* = 4.09, *df*1 = 3, *df*2 = 788, *p* = .33, self-disclosure to unknown online friends: *F* = 2.98, *df*1 = 3, *df*2 = 788, *p* = .12), the results showed that students’ Department of studies (Departments of Schools of Humanities and Social / Polytechnic / Economic Sciences) did not significantly affect neither their intensity of Facebook use (*p* > .05) nor their self-disclosure to unknown online friends (*p* > .05).

Meeting the assumptions of Levene’s Test of Equality of Error Variances for both Facebook behaviors (intensity of use: *F* = 2.19, *df*1 = 3, *df*2 = 788, *p* = .28, self-disclosure to unknown online friends: *F* = 3.21, *df*1 = 3, *df*2 = 788, *p* = .32), students’ level of studies (undergraduate/postgraduate) seemed to significantly affect their intensity of online use, *F*(1, 290) = 9.873, *p* = .002, partial *η*^2^ = .39, and their self-disclosure to unknown online friends, *F*(1, 290) = 10.201, *p* = .001, partial *η*^2^ = .31. In particular, undergraduate compared to postgraduate students seemed to make more intense Facebook use (Mean = 4.01, SD = .82 and Mean = 3.45, SD = .53, respectively) and self-disclosure to unknown online friends (Mean = 3.15, SD = 0.48 and Mean = 2.73, SD = .55, respectively).

Finally, meeting the assumptions of Levene’s Test of Equality of Error Variances for both Facebook behaviors (intensity of use: *F* = 4.31, *df*1 = 3, *df*2 = 788, *p* = .48, self-disclosure to unknown online friends: *F* = 2.11, *df*1 = 3, *df*2 = 788, *p* = .23), the results showed that students’ gender did not significantly affect neither their intensity of Facebook use (*p* > .05) nor their self-disclosure to unknown online friends (*p* > .05).

## Discussion

The present study aimed to investigate the collective pattern of Facebook use by university students (intensity of its use self-disclosure to unknown online friends) during the period of the COVID-19 pandemic, examining at the same time the role of loneliness and resilience.

According to the descriptive findings, university students during the period of the COVID-19 pandemic seemed to make intense Facebook use. Specifically, most students (69%) mentioned that they spent more than 3 h per day on Facebook and admitted their emotionally over-engagement in Facebook activities generally. At the same time, students admitted that to a great extent reveal feelings and personal information to unknown online friends. This was expected based on Hypothesis 1 and it is in-line with other previous similar studies mentioning that university students tend to spend many hours daily on Facebook for entertainment, implying a possibly timely excessive way of its use (Junco 2012; Locatelli et al. 2012; Michikyan et al. 2015; Sun 2020; Tandoc Jr et al. 2015). Also, the above finding reflects the long use of Facebook by adults during and in the aftermath of the pandemic period (Eghtesadi and Florea 2020; Erliza and Septianingsih 2022; Indriawati and Wibowo 2021; Raamkumar et al. 2020; Wiederhold 2020). The severe modifications in many academic processes (e.g., distant meetings, presentations, and exams) that are still valid in many universities due to the different epidemiological circumstances in different countries (Bourdas and Zacharakis 2020; Erliza and Septianingsih 2022; Galanis et al. 2020), have probably created a fertile ground for students to consider social networking sites, such as Facebook, as an integral and possibly helpful part of their daily social and academic life.

However, within this context it seems that students often turn up to timely over-use Facebook and share personal (possibly) sensitive information with unknown online friends, as they expressed a relatively high (more than average) intensity of Facebook use as well as a relatively high (more than average) self-disclosure to unknown online friends. Although the high perceived intensity of Facebook use refers to the students’ numerous and emotional interactions, not exclusively with unknown, but with known online friends as well, this finding requires further attention, as it reflects youths’ tendency to be timely and emotionally over-involved in Facebook environment. This finding along with the students’ tendency to make self-disclosures to unknown online friends regarding personal sensitive information (e.g., deep feelings, thoughts, and plans) could reflect the following: a possibly less safe collective pattern of Facebook use especially if we consider that the intense use of social networking sites enhance the likelihood of being cyberbullied by both known and unknown online friends (Barlett et al. 2018; Dredge et al. 2014; Rachoene and Oyedemi 2015; Touloupis 2022).

Also, the descriptive findings showed that the participating students expressed a relatively high sense of loneliness and a moderate sense of resilience, reflecting young adults’ negative emotions since the outbreak of the COVID-19 pandemic (Ernst et al. 2022; Groarke et al. 2020; Hwang et al. 2020; Rosenberg 2020; Touloupis 2021; Touloupis and Athanasiades 2022; Vinkers et al. 2020; Zhang et al. 2022). It is likely that the significant and sudden changes in university students’ daily routine during the last two years of the COVID-19 pandemic have caused a collectively more vulnerable emotional state among youth.

Additionally, the results of the path analyses model revealed that students’ sense of loneliness predicts in a positive way their self-disclosure to unknown online friends not only directly but also indirectly, through the positive mediating role of their perceived intensity of Facebook use. This finding was expected based on Hypothesis 2a. Also, it is in-line with other studies, which mention that young adults who feel lonely tend to use internet and social networking sites (e.g., Facebook) in a problematic way (Alheneidi et al. 2021; Costa et al. 2019; Κim et al. 2009). Therefore, it is likely that students who feel lonely during the current period may succumb to the temptation to self-disclose personal and sensitive information to unknown online friends. This temptation seems to be pronounced in the case of lonely students who develop a pattern of intense Facebook use (engage with various interactive online activities such as posting, sharing, and chatting).

Furthermore, the study showed that students’ sense of resilience negatively moderates the relationship between their sense of loneliness and their intensity of Facebook use. This finding was expected based on Hypothesis 2b and it is consistent with other related studies (Alheneidi et al. 2021; Bilgin and Tas 2018; Costa et al. 2019; Dimogiorga and Sygkollitou 2015; Κim et al. 2009; Nam et al. 2018; Tidwell and Walther 2002; Valkenburg and Peter 2009; Yen et al. 2019). It seems that students’ sense of resilience has the potential to weaken the predictive relationship between students’ sense of loneliness and their intense Facebook use (Bilgin and Tas 2018; Nam et al. 2018; Yen et al. 2019), which in turn increases their likelihood to make self-disclosures to unknown online friends (Dimogiorga and Sygkollitou 2015). Therefore, students who feel lonely but resilient are less prone to make intense Facebook use and possibly succumb to the temptation to reveal personal sensitive information to unknown online friends. This implies that strengthening sense of resilience of young adults who feel lonely during crisis periods, such as the COVID-19 pandemic, could act as a protective filter against the development of an intense and possibly less safe pattern of Facebook use. This necessity is even more pronounced considering the relatively low sense of resilience among the participating students as well as the generally vulnerable emotional state (high sense of loneliness and low sense of resilience) of young adults during and in the aftermath of the COVID-19 pandemic (Ernst et al. 2022; Groarke et al. 2020; Hwang et al. 2020; Rosenberg 2020; Touloupis 2021; Touloupis and Athanasiades 2022; Zhang et al. 2022).

Regarding students’ demographic/personal characteristics, the analyses of variance showed that students’ university department of studies did not seem to significantly affect neither their intense Facebook use nor their self-disclosure to unknown online friends. Although students from specific Departments (e.g., Psychology/Education) have been taught courses (e.g., Educational/School/Clinical Psychology), which address issues of risky/unsafe online behaviors (Guides of Undergraduate/Postgraduate Studies, Department of Psychology/Education, University of Western Macedonia, 2021–2022), and are expected to be more sensitized regarding the issue studied, compared to students from other Departments (e.g., Polytechnic School), this was not the case for the present study. It could be supposed that the circumstances during the last two years of the pandemic period (Erliza and Septianingsih 2022; Rogers et al. 2021) have made all university students, regardless the field of their studies, to use Facebook as a way to compensate for their limited face-to-face interactions, resulting in that way in a possibly maladaptive pattern of Facebook use (intense use with self-disclosures to unknown online friends).

On the contrary, the analyses of variance regarding students’ level of studies (undergraduate / postgraduate) showed that this personal characteristic seemed to significantly affect students’ pattern of Facebook use. Particularly, it was found that the undergraduate students seemed to make more intense Facebook use and self-disclosure to unknown online friends, compared to the postgraduate students. Since most of the participating undergraduate students were in the second year of their studies, this could mean that they are closer to late adolescence, where it is usually reported an excessive use of social media (Dhir and Tsai 2017; Marino et al. 2016; Stockdale and Coyne 2020). Therefore, undergraduate students and mainly those at the beginning of their academic life (beginning of the second year of studies) could be more prone to spend many hours on Facebook daily, especially in the current period, compared to older young adults such as postgraduate students. Also, the fact that for undergraduate students the transition from school to academic life and their new socio-interpersonal acquaintances with fellow students were suddenly interrupted by the COVID-19 pandemic could possibly explain why they seem more prone to develop a maladaptive pattern of Facebook use (intense use, self-disclosure to unknown online friends), to make up for their “lost” academic years and socio-interpersonal relationships. Furthermore, considering that postgraduate students usually attend more demanding academic programs (e.g., Master, Doctoral, and Postdoctoral) and obligations, compared to undergraduate programs, could explain why the report a less maladaptive pattern of Facebook use.

Finally, the analyses of variance regarding students’ gender showed that intensity of Facebook use and self-disclosure to unknown online friends concern both men and women students. This finding was expected according to Hypothesis 3. Also, it is consistent with previous studies, which conclude that both men (Cudo et al. 2020; Durndell and Haag 2002; Joiner et al. 2005; Mazman and Usluel 2011; Muscanell and Guadagno 2012; Raacke and Bonds-Raacke 2008), and women (Farinosi and Taipale 2018; Peter et al. 2005; Petronio 2002; Rose and Rudolph 2006; Sheldon 2013) spend a lot of time on Facebook and reveal personal sensitive information (self-disclosure) trying to make new online acquaintances. It is likely that the limited social interactions during the last two years of the pandemic period have made youths, regardless of gender, more oriented toward the long use of Facebook and less cautious toward the development of online acquaintances with unknown people (Eagly 1987).

### Limitations, future research and contribution of the study

The findings of the present study should be interpreted with caution as they are subject to specific limitations. For example, the possibly students’ socially acceptable responses to the questionnaire, the selection of the sample from Greek universities and the focus on a specific social media platform may limit the validity and the generalizability of the data. Also, the study followed a quantitative method, which did not allow for in-depth qualitative investigation of the students' relevant experiences. Furthermore, the unequal distribution of students, based on the level of their studies, did not allow us to examine whether their pattern of Facebook use is differentiated by the year of undergraduate studies, or the postgraduate program attended (MSc, PhD, Postdoc). Finally, the cross-sectional research design of the study cannot conclude to safe casual relationships among the variables involved.

These limitations could trigger future relevant transnational longitudinal studies to strengthen the generalizability of the results and confirm the proposed structural model that explains university students’ pattern of Facebook use the current period. Additionally, a future study could examine whether this structural model applies in the case of using other social media platforms (e.g., Instagram, Twitter, and TikTok) or if different directions are observed in the structure of the relationships between the variables involved (e.g., investigating the possible negative predictive role of resilience in sense of loneliness or the negative predictive role of intense Facebook use in sense of loneliness). Furthermore, a larger sample of university students would allow the investigation of the statistical effects mentioned before. Finally, a complementary qualitative investigation of the issue under study, through individual semi-structured interviews or focus groups with students, could capture more qualitative aspects of their emotional state and their pattern of Facebook use.

Nevertheless, the present study contributes to international related literature. It constitutes a first attempt to depict the structure of the relationships between emotional (loneliness, resilience) and behavioral factors (intensity of Facebook use) to propose an explanatory model of university students’ tendency to make self-disclosures to unknown online friends. This is of high importance considering that in many countries several academic procedures in tertiary education still run online or in a hybrid way influencing students’ daily academic life (Erliza and Septianingsih 2022; Zrnić Novaković et al. 2022). The proposed explanatory model of the present study constitutes a significant source of information and awareness for professionals of mental health, such as school, educational and/or counselling psychologists working at university campuses (Seidel et al. 2020). The findings could imply the importance of launching or enriching university counselling actions (Belskaya et al. 2016; Bitsios et al. 2017; Seidel et al. 2020) to enhance students’ prudent use of social media especially during unstable and crisis periods, such as the pandemic period. At the same time, within these actions interpersonal relationships with peers could be strengthened, especially in the case of the first-year undergraduate students who experience their first transitional period from school to academic life. This could be achieved by establishing, for example, frequent voluntary and experiential online, hybrid or face-to-face activities/workshops, that focus on students’ sense of personal adequacy and their positive adaptation to possible difficult life situations/changes especially amid crisis periods. Weakening in this way students’ sense of loneliness and strengthening their sense of resilience could make them less vulnerable to engage with a possibly less safe pattern of social media use. These prevention actions may be more beneficial especially for university students who belong to vulnerable groups, due to health problems related to the COVID-19 pandemic, and they are officially entitled to abstain from university campuses (Drane et al. 2020). Undoubtedly, the frequency and quality assurance of the above proposed measures presupposes that the university counselling centers will be able to provide enough mental health specialists within campuses.

## Conclusions

In summary, the present study concludes that during unstable periods, such as amid and in the aftermath of the COVID-19 pandemic, a collective maladaptive pattern of Facebook use seems to be reflected among male and female undergraduate university students, as they tend to make intense Facebook use and they easily reveal personal sensitive information to unknown online friends (self-disclosure). Finally, students’ emotional mechanisms, such as their sense of loneliness and resilience seem to have the potential to act as a risk and protective factor respectively, explaining the development of their pattern of Facebook use.

## Data Availability

Data not available (Due to the sensitive nature of the research topic, the participating students were assured raw data and material would remain confidential and would not be shared).
